# Intercostal nerve cryoablation for control of pain after sternal fracture repair: A case series

**DOI:** 10.1016/j.tcr.2026.101369

**Published:** 2026-05-20

**Authors:** Laurel Foster, Colin L. Doyle

**Affiliations:** a Texas A&M Health Science Center - Round Rock, 3950 N A.W. Grimes Blvd, Round Rock, TX, 78665, United States of America; b St. David's Round Rock Medical Center, 2400 Round Rock Ave, Round Rock, TX, 78681, United States of America

**Keywords:** Intercostal cryoablation, Intercostal cryoneurolysis, Sternal fracture, Sternal fixation, Sternal fracture pain, Sternal fracture analgesia, Traumatic sternal fracture

## Abstract

**Introduction:**

Sternal fractures resulting from blunt chest trauma, can present with significant pain and respiratory compromise, particularly in patients with polytrauma. While isolated, nondisplaced fractures are typically managed conservatively, displaced or painful fractures are higher risk and may warrant surgical intervention. Intercostal cryoneurolysis (IC) has emerged as a promising adjunct, offering enhanced pain control in thoracic procedures. However, no studies have characterized the role of IC in the management of traumatic sternal fractures.

**Case report:**

We present two cases of patients with displaced sternal fractures following motor vehicle collisions who underwent surgical fixation with adjunctive IC. Both patients reported severe preoperative sternal pain unrelieved by multimodal analgesia. Intraoperative IC was performed bilaterally at the level of the fracture and adjacent intercostal spaces using a cryoanalgesia probe. Postoperatively, both patients experienced immediate and sustained pain relief, with complete insensate status over the sternal region and no complications. One patient was discharged the day after surgery and resumed normal activities, while the other remained hospitalized until postoperative day 7 for management of associated orthopedic injuries.

**Discussion:**

These cases demonstrate that IC, when used alongside sternal fixation, can provide rapid, effective, and durable pain relief, potentially reducing reliance on opioids and facilitating early mobilization. While further research is needed to establish standardized protocols, these findings support the integration of IC into the surgical management of traumatic sternal fractures.

## Highlights

• The first two reported cases of intercostal cryoablation performed for sternal fracture fixation.
• Intercostal cryoablation provides additional analgesia when fixating sternal fractures.
• Intercostal cryoablation can minimize need for narcotic medications.

## Introduction

Fractures of the sternum commonly result from blunt chest trauma such as a direct blow to the anterior chest wall or forced deceleration as seen in MVAs and falls [1]. Clinical features include moderate to severe chest pain and tenderness often exacerbated by coughing or sneezing, difficulty breathing, and overlying ecchymoses [1]. Sternal fractures can range from minor to severe. Isolated, nondisplaced fractures are usually low-risk, while those with multiple associated injuries carry a higher risk of complications and death [2], [3].

**Fig. 1:**
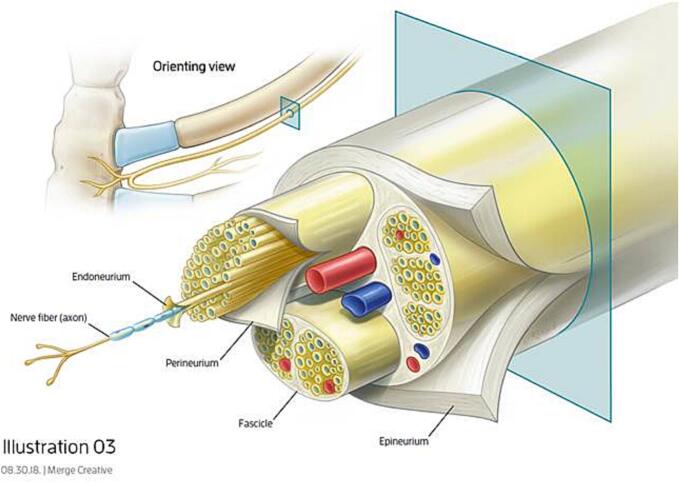
Diagram showing anatomic relationship between intercostal nerves and sternum as well as the components of the intercostal nerve.

Intercostal cryoneurolysis (IC) has shown effectiveness in reducing pain when used as an adjunct to surgical stabilization of rib fractures [4], pectus excavatum [5], and pulmonary resection [6]. Cryoneurolysis induces breakdown of the nerve's axon and myelin sheath, triggering Wallerian degeneration in the portion of the nerve beyond the treatment site. This effectively causes numbness distal to the treatment site which is long-acting, but temporary due to the endometrium, perineurium and epineurium remaining intact, allowing for nerve regeneration [7] (Fig. 1). Presented here are two cases, Patient 1 and Patient 2, of successful control of pain associated with sternal fractures with adjunctive IC combined with fracture fixation.

## Case report

### Patient 1

**Fig. 2:**
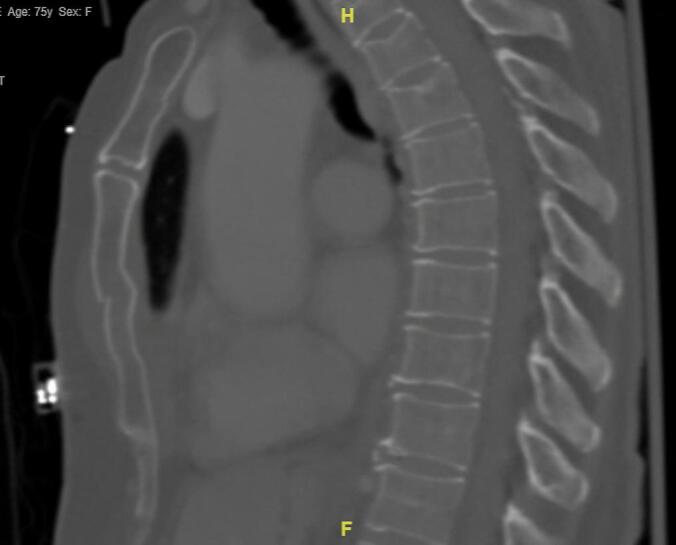
Preoperative CT scan demonstrating displaced sternal fracture in Patient 1.

Patient is a 75-year-old female with a history of hypertension, hyperlipidemia, and arthritis status post total knee arthroplasty. She presented to the Emergency Department (ED) after a motor vehicle crash at highway speed. She had an obvious open fracture of the left ankle and was endorsing chest pain with respiration. She was evaluated by the Trauma Team following ATLS protocols. Exam and imaging revealed a displaced sternal fracture (Fig. 2) in addition to bilateral rib fractures, a grade II liver injury, a complex fracture of the left medial malleolus and fibula. She was administered antibiotics and tetanus prophylaxis. The patient was brought to the operating room urgently with Orthopedic Surgery for washout and placement of an external fixator on the left leg and ankle fractures.

On further evaluation of the patient's chest wall injury she reported severe pain to the sternal region with only minimal pain from the rib fractures. This pain was present despite being placed on multimodal analgesic regimen per our institution's guidelines for chest wall injury. She endorsed popping and clicking in her central chest with respiration. On exam she had paradoxical movement of the upper and lower portions of the sternum with the respiratory cycle. We discussed sternum fixation with cryoablation of the intercostal nerves as well as rib fixation. Because the sternal fracture was the primary source of her pain we decided to address this injury first and then assess the severity of her rib pain and need for further intervention.

On hospital day 2, the patient was brought to the operating theater for sternal fracture fixation with intercostal cryoablation. The patient was placed supine with arms tucked and a rolled blanket between the scapulas to help expand the anterior chest. The fracture was localized with ultrasound using a linear probe, and a limited sternotomy incision was made. The anterior sternum was exposed and the fracture reduced with a blunt right-angle clamp. The fracture was fixated using a titanium 16-hole ladder plate (Zimmer-Biomet, Sternalock Blu, Jacksonville, FL, USA). Once this was completed and the construct assessed for stability, the surface of the rib at the level of the fracture, as well as one rib space cephalad and one rib space caudally to the fracture site were bluntly cleared of connective tissue.

**Fig. 3:**
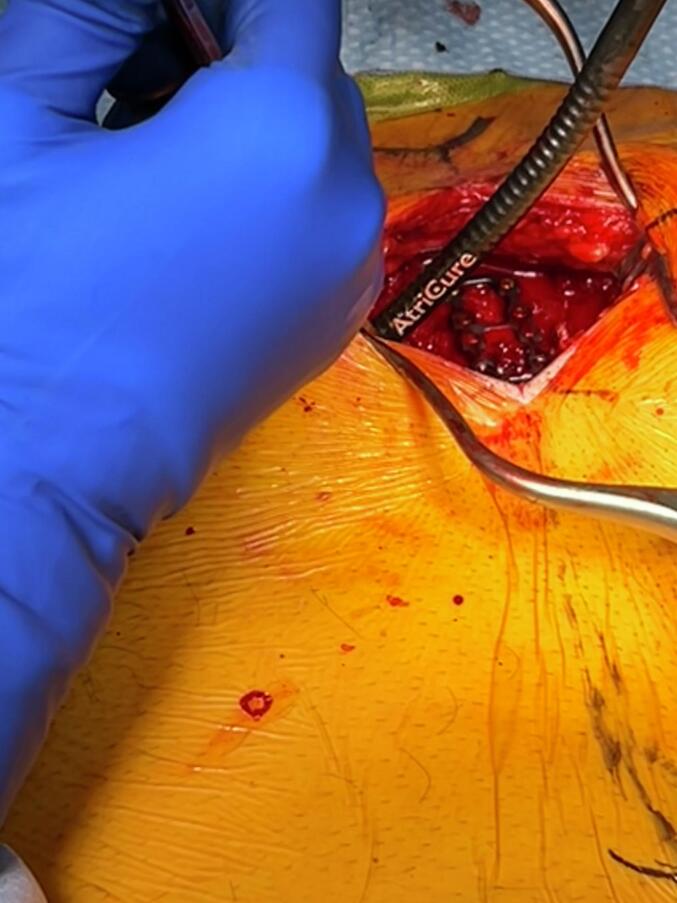
Intraoperative cryoneurolysis of intercostal nerves using cryoSPHERE MAX probe.

We then performed cryoneurolysis of the intercostal nerves at the level of the fracture, as well as one level cephalad and one level caudal to the fracture site. This was performed using a cryoanalgesia probe (cryoSPHERE MAX Probe, Atricure, Mason, OH, USA). The probe was placed at least 2 cm lateral to the sternum on the cephalad aspect of the intercostal space, on the exterior surface of the intercostal muscles. During cryoneurolysis an appendiceal retractor was used to ensure no tissues were touching the probe other than the structures of the intercostal space (Fig. 3). The freezing cycle was performed for 60 s at each nerve level. We then performed the same procedure on the opposite side, including the blunt detection and cryoneurolysis at the same intercostal levels. The wound was closed in multiple layers and the patient was brought to the recovery room.

While in the recovery room the patient reported immediate improvement in her symptoms of pain and resolution of the clicking sensation. By postoperative day 1, she was insensate to the sternal area with minimal pain from her sternal fracture or incision. She continued to endorse minimal pain from rib fractures and therefore these were treated non-operatively. The patient remained in the hospital for internal fixation of her extremity fractures after resolution of swelling and was discharged to home on postoperative day 7.

The patient was seen in clinic for follow up on postoperative day 26. She continued to note minimal pain from her sternal fracture and on exam continued to be insensate to the sternal area. No postoperative complications were noted and the sternum remained stable.

### Patient 2

**Fig. 4:**
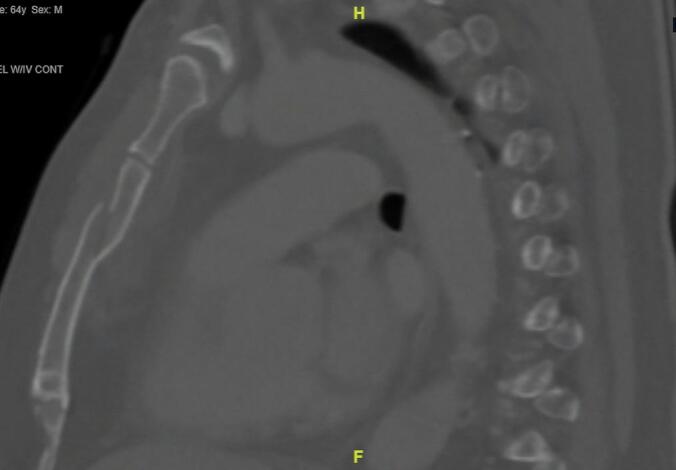
Preoperative CT scan demonstrating displaced, oblique sternal fracture in Patient 2.

Patient is a 64-year-old male with a history of hypertension and obstructive sleep apnea who presented to the emergency room after a motor vehicle crash at moderate speed. He was complaining of neck and chest pain and was initially evaluated by the Emergency Department staff. On imaging, the patient was noted to have a fracture of the transverse process of the C7 vertebrae and a left internal carotid artery dissection flap consistent with a Biffl grade II injury. CT imaging of the chest demonstrated a displaced, oblique sternal fracture (Fig. 4). The Trauma Team was then consulted for evaluation. Neurosurgery was also consulted and ordered an MRI of the cervical spine and started the patient on aspirin 81 mg daily.

On evaluation by the Trauma Team, the patient complained of severe pain to the sternum with any movement despite receiving multimodal analgesia. He denied clicking and did not have paradoxical chest wall movement on exam. Sternal fixation with intercostal cryoablation was discussed with the patient, and due to the severity of his pain the patient decided to proceed with this surgical intervention.

On hospital day 2, the patient was brought to the operating room for sternal fixation and intercostal cryoablation. He was positioned in a similar fashion and the dissection and fixation was performed in a similar manner using a titanium 16-hole ladder plate (Zimmer-Biomet, Sternalock Blu, Jacksonville, FL, USA). The cryoneurolysis was also performed in a similar manner, using the cryoanalgesia probe (cryoSPHERE MAX Probe, Atricure, Mason, OH, USA) for a 60 s cycle at the level of the fracture as well as one level cephalad and one level caudal to the fracture level. This was performed bilaterally. The patient was brought to the recovery room.

While in the recovery room the patient noted improvement in his symptoms. By postoperative day 1 he was insensate to the sternal region and reported excellent pain control. He was evaluated by our therapists and was discharged to home on postoperative day 1. The patient presented to clinic for follow-up on postoperative day 12 and continued to be insensate to the sternal region on exam. He reported only minimal symptoms when performing overhead movements. He also reported having travelled several hours via airplane to attend his child's college graduation, speaking to the adequacy of his pain control.

## Discussion

Intraoperative IC cryoablation was originally described in 1974 as a means to significantly reduce thoracotomy pain [8]. Notably, IC cryoablation improves ability to have good respiratory effort, reduces the need for repetitive nerve blocks, and mitigates adverse effects of long-term opioid medications [8]. For management of traumatic rib fractures, intraoperative IC has been shown to be an effective pain management modality with reduced hospital costs, shorter hospital and ICU stays and decreased narcotic use [9]. Likewise, IC has shown benefit in other chest wall procedures such as pectus excavatum repair [5] and pulmonary resection [6]. While additional long-term research is necessary, initial findings indicate that chest wall sensation typically returns and persistent neuropathic pain is uncommon [10].

Sternal fractures are often viewed as benign injuries that can be managed with conservative measures such as rest and analgesia [1], [11]. However, indications for sternal fracture fixation are becoming better delineated. Surgical fixation should be considered in the setting of fracture instability, significant displacement, uncontrolled pain, and respiratory compromise, or chronically in the setting of fracture non-union [1]. Compared to nonoperative management, surgical fixation of sternal fractures leads to enhanced respiratory recovery and decreased reliance on analgesics, though there is no attributed difference in pain scores, intensive care unit length of stay, or hospital length of stay [12]. Intercostal nerve cryoablation shows promise in improving analgesic management of chest wall trauma patients.

These cases expand on current literature regarding the management of traumatic rib fractures with intraoperative IC. In these two patients with traumatic sternal fractures, IC provided rapid and effective pain relief without any reported adverse effects. While further research is needed to establish best practices and guidelines for IC use in this population, these cases highlight its strong potential as a tool for reducing pain after sternal fracture fixation. Given that many chest wall injuries are polytraumatic and come with substantial comorbidity, intercostal nerve cryoablation offers a promising approach to enhancing pain control and outcomes across patients who present with varying severities and combinations of injuries.

## CRediT authorship contribution statement

**Laurel Foster:** Writing – review & editing, Writing – original draft, Investigation, Formal analysis. **Colin L. Doyle:** Writing – review & editing, Writing – original draft, Supervision, Project administration, Data curation, Conceptualization.

## Declaration of competing interest

The authors declare that they have no known competing financial interests or personal relationships that could have appeared to influence the work reported in this paper.
